# Rescue permanent LVIS stenting with post-stenting angioplasty after failed mechanical thrombectomy for refractory internal carotid artery occlusion at the paraclinoid segment: two-case report

**DOI:** 10.1186/s41016-020-00221-1

**Published:** 2021-01-11

**Authors:** He Li, Zifu Li, Weilong Hua, Yongxin Zhang, Wenjin Yang, Mingtao Feng, Lei Zhang, Pengfei Xing, Yongwei Zhang, Bo Hong, Pengfei Yang, Jianmin Liu

**Affiliations:** 1 Stroke Center, Changhai Hospital, Navy Medical University, Shanghai, China; 2 Graduate School, Navy Medical University, Shanghai, China; 3 Department of Neurosurgery, Changhai Hospital, Navy Medical University, Shanghai, China; 4 Department of Neurosurgery, People’s Hospital of Pudong New Area, Shanghai, China

**Keywords:** Large vessel occlusion, Stent retriever, Stent, LVIS

## Abstract

**Background:**

Previous studies indicated the effectiveness of permanent stenting when dealing with retriever-failed refractory large vascular occlusion (LVO). Variety types of stents were implanted permanently to achieve recanalization. Low-profile visualized intraluminal support (LVIS) is generally used as a supportive device for embolization of intracranial aneurysm. Its specific structural and functional characteristics contribute to its potential of treating LVO.

**Cases presentation:**

A 51-year-old male was transferred to our stroke center because of conscious disturbance with the weakness of the left upper limb. The National Institute of Health Stroke Scale (NIHSS) was 24; the Glasgow Coma Scale (GCS) was 10. Digital subtraction angiography (DSA) showed that his paraclinoid segment of R-ICA was occluded due to hard clot embolization. Thrombectomy was performed 6 times, but the occlusion remained. Finally, LVIS was implanted permanently and post-dilation was performed, which successfully recanalized the artery (eTICI 2c). The post-operative NIHSS and GCS were 20 and 11, respectively, which were 10 and 14 when discharged.

Another patient was a 71-year-old male who suffered weakness of left limbs. NIHSS was 15; GCS was 11. DSA confirmed that the paraclinoid segment of his R-ICA was occluded due to hard clot embolization. Totally 6 times of mechanical thrombectomy, angioplasty, and tirofiban infusion were performed, which failed to recanalize the artery. In the end, LVIS implantation with post-dilation was performed, and full recanalization was achieved (mTICI 3). The post-operative NIHSS and GCS were 9 and 15, respectively, which were 3 and 15 when discharged.

**Conclusions:**

These 2 cases invited LVIS into the treatment of refractory occlusion due to hard clot embolization at the paraclinoid segment, and the outcomes were preferable because of the higher visibility, higher flexibility, and lower cell size of LVIS.

## Background

Previous RCTs showed about a 30% failure rate of mechanical thrombectomy in patients with intracranial large vascular occlusion (LVO) [1]. Refractory occlusion is one of the leading causes of failed thrombectomy. Currently, permanent stenting is one of the effective ways to deal with refractory occlusion [2, 3]. However, there remained many cases, in which the recanalization could not be achieved because of hard embolus obstruction or large clot burden. The hard emboli tended to protrude into the lumen and led to re-occlusion due to the big cell size of stents that commonly implanted in refractory occlusion, such as Wingspan, Enterprise, and Solitaire. Thus, the stent selection strategy still needs to be optimized.

Low-profile visualized intraluminal support (LVIS) device is a self-expanding nickel-titanium stent. It is a single-wire braided, closed-cell, re-sheathable intracranial stent originally designed for stent-assisted coil embolization [4]. It has two radiopaque wires, which makes it easily visible, and the size of its cells is small due to the high percentage of metal coverage. These characteristics contribute to its great potential to rescue retriever-failed refractory LVO due to hard clot embolization.

Our case report showed 2 cases that used LVIS implantation as rescue therapy after several times of thrombectomy. All the patients in these cases achieved eTICI 2c-3 recanalization in the end after endovascular treatment.

## Case presentation

### Case 1

**Fig. 1:**
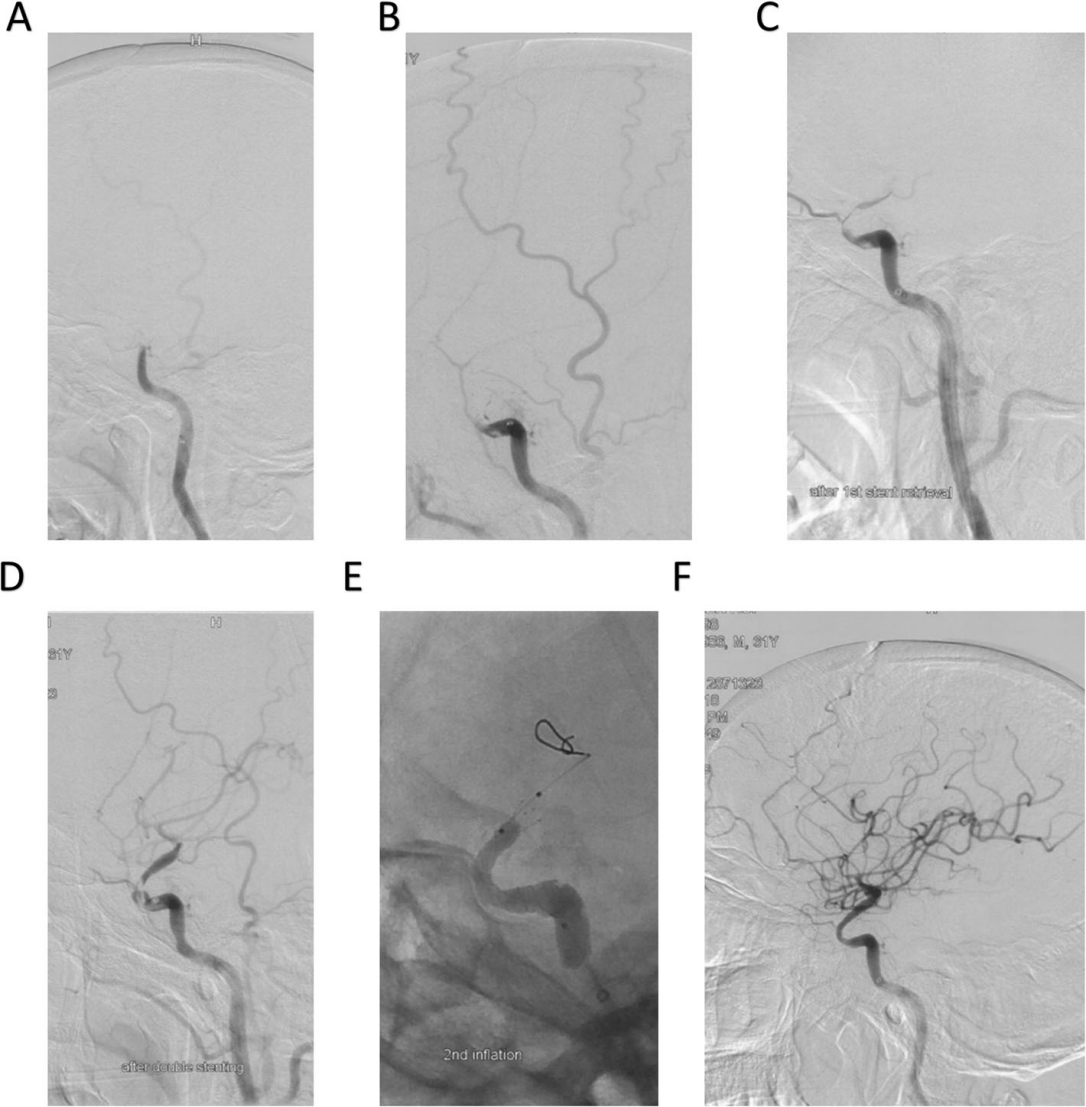
The endovascular treatment procedure of case 1. **A** The occlusion site. **B** The occlusion remained after ADAPT. **C**, **D** The occlusion still remained after several times of thrombectomies. **E** The procedure of LVIS implementation. **F** The final result of reperfusion

A 51-year-old Asian male with a history of hypertension and dissection of aorta suddenly got a conscious disturbance with weakness of left upper limb (muscle force level was 0) and paraplegia of lower limbs 13 days after cardiac surgery of aortic arch replacement with stent implantation at descending aorta (Table 1). The National Institute of Health Stroke Scale (NIHSS) was 24, and the Glasgow Coma Scale (GCS) was 10 (E2V3M5). Multimodal CT showed the right internal carotid artery (ICA) occlusion and a hypoperfusion of the right hemisphere. The cerebral blood flow (CBF) < 30% volume was 28 ml, Tmax > 6.0 s volume was 195 ml, and the mismatch volume was 167 ml. Digital subtraction angiography (DSA) confirmed the occlusion was at the paraclinoid segment of R-ICA, and endovascular treatment was decided to be performed (Fig. 1A). An 8F MPA-1 (Johnson & Johnson Cordis, USA) guiding catheter was positioned at the cervical segment of R-ICA. A 6F Sofia plus (MicroVention Terumo, USA) catheter supported by microwire and a microcatheter were advanced to the occlusion site of the paraclinoid segment. A direct aspiration first-pass technique (ADAPT) was performed, but the occlusion remained (Fig. 1B). Stent-retrieval thrombectomy combining with direct aspiration (Solumbra technique) [5] was performed with Solitaire FR (Dendron, acquired by ev3, Inc. USA) (6 × 30 mm) for 3 times, but full recanalization still could not be achieved (Fig. 1C). Parallel Y-stent thrombectomy strategy was then performed with Solitaire FR (6 × 30 mm) and Solitaire FR (4 × 20 mm) for 2 times, which also failed to recanalize the occlusion (Fig. 1D). Thus, LVIS stent (MicroVention Terumo, USA) (3.5 × 15 mm) was deployed at the occlusion site, and the microcatheter was advanced through the LVIS along the delivery system. An exchange guidewire was advanced through the microcatheter, and a Scepter C balloon (MicroVention Terumo, USA) (4.0 × 15 mm) was exchanged. Angioplasty was performed by the balloon under 8 atm (Fig. 1E). The final angiography showed that an eTICI 2c recanalization was achieved (Fig. 1F).

**Table 1:** The baseline characteristics and the outcomes of the two cases

	Case 1	Case 2
Age	62	71
Sex	Male	Male
GCS	10	11
NIHSS	24	15
Onset to admission (min)	220	277
Onset to multimodal CT (min)	263	297
Onset to puncture (min)	330	360
Onset to recanalization (min)	625	669
Procedure time (min)	295	309
Post-operative eTICI	2c	3
Post-operative GCS	11	15
Post-operative NIHSS	20	9
GCS when discharged	14	15
NIHSS when discharged	10	3
Six-month mRS	4	2

The post-operative GCS 24 h was 11 (E3V3M5), the NIHSS was 20, and the strength of left upper limb became level 1. The patient was then transferred to the intensive care unit of the department of cardiac surgery. Clopidogrel 75 mg/day by gastric tube and tirofiban (100 ml, 5 mg) 10 mg/day intravenously were administrated for 1 week, and then, the anti-platelet strategy was changed into aspirin 100 mg/day and clopidogrel 75 mg/day. The patient was discharged 1 month later, but he still suffered paraplegia because of the spinal reperfusion injury in aortic arch replacement. His GCS was 14 (E4V5M5), and NIHSS was 10 when he was discharged, and his 6-month modified Rankin Scale (mRS) scored 4 because of the lower limb (muscle force level was 0) and left upper limb (muscle force level was 2) disability.

### Case 2

**Fig. 2:**
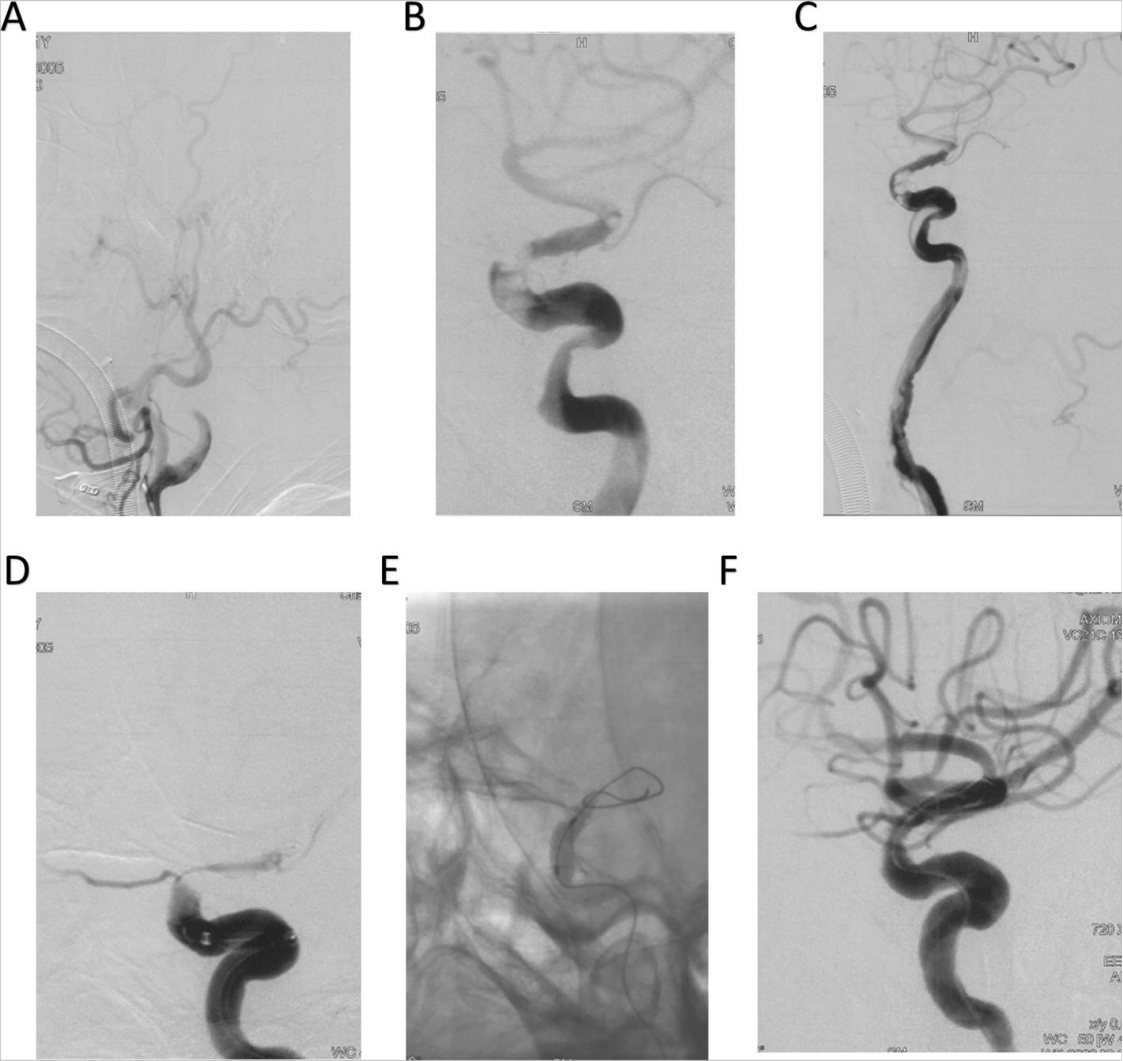
The endovascular treatment procedure of case 2. **A** The occlusion site. **B** The occlusion remained after 4 times of thrombectomy. **C** Gateway angioplasty did not fully recanalize the occluded artery. **D** The occlusion still remained after double-stent thrombectomy. **E** The procedure of LVIS implantation. **F** The final result of reperfusion

A 71-year-old Asian male was transferred to the stroke center because of the weakness of left limbs (muscle force level was 0) for 5 h. Physical examination showed that his GCS was 11 (E3V3M5) and NIHSS was 15 (Table 1). Multimodal CT showed an occlusion at the right ICA and a hypoperfusion of the right hemisphere. The Tmax > 6.0 s and CBF < 30% volume were 66 ml and 0 ml, respectively, and the mismatch volume was 66 ml. DSA confirmed the occlusion site was at the paraclinoid segment R-ICA, and endovascular treatment was decided to be performed (Fig. 2 A). Thrombectomy was first performed with ADAPT for two times but failed. Thus, microcatheter was navigated to the M1 segment, and the third time of mechanical thrombectomy was performed using the “Solumbra” technique with Solitaire FR (6 × 30 mm). However, the paraclinoid segment of R-ICA remained occlusive. Another twice of the “Solumbra” technique also failed (Fig. 2 B). So, angioplasty was performed with a Gateway balloon catheter (Stryker, USA) (3.0 × 15 mm) at the occlusion site, followed by a 12-ml tirofiban (100 ml, 5 mg) bolus and continued infusion (8 ml/h). But re-occlusion was observed 15 min after tirofiban infusion (Fig. 2C). Another 3 times of mechanical thrombectomies with double-stent technique were performed, but we only pulled out part of the thrombus (Fig. 2D). In the end, LVIS (4.5 × 30 mm) was implanted permanently to the occlusion site, and post-stenting angioplasty was performed with Gateway balloon (4.0 × 9.0 mm) under 8 atm (Fig. 2E). The final angiography showed that an eTICI 3 recanalization was achieved after the operation (Fig. 2F).

His NIHSS became 9, GCS became 15, consciousness was significantly improved, and the muscle strength of left limbs was three 24 h after operation. He was routinely administrated aspirin 100 mg/day with clopidogrel 75 mg/day orally for 1 month, and aspirin 100 mg/day for lifelong. His NIHSS was 3 when he was discharged 17 days after operation, and his mRS scored 2, 6 months after the operation.

## Discussion

As far as we know, this is the first report about permanently implanting LVIS to deal with embolic refractory intracranial LVO. Refractory occlusions, which remained after several times of thrombectomy and angioplasty, happened in both two cases, and they both occurred at the paraclinoid segment of ICA. The leading cause of this phenomenon might be the tortuous shape of the paraclinoid segment made the embolus easier to be blocked at this segment. These blocks are usually hard clots, which are difficult to be pulled out. In both cases, LVIS implantation with post-stenting balloon dilation was used as the final therapy and mTICI 2b-3 grade recanalization was achieved finally, which improved the neurological scores and symptoms of the patients. These results primarily conveyed the feasibility of LVIS implantation in dealing with refractory intracranial LVO.

Permanent stent implantation with or without angioplasty is a preferable rescue therapy for unsuccessful recanalization. The efficacy of this technique has been generally accepted by practitioners, and it is performed more actively these years to deal with refractory LVO. Different types of stents, including Solitaire stent, Apollo stent, Enterprise stent, Wingspan stent, and Neuroform stent, have been implanted to deal with refractory occlusion [1–3, 6]. However, in the case of hard clot embolization, the remaining hard clots may protrude into these large-cell stents, which will increase the risk of re-stenosis and thrombosis.

Thus, to explore a novel effective strategy specifically for refractory LVO due to hard clot embolization, we creatively used LVIS, which is originally designed for treating intracranial aneurysms. In our cases, more than 3 times of thrombectomy and other techniques were used but failed, and an LVIS implantation with post-stenting angioplasty finally achieved recanalization. We supposed that if we used this technique in advance, the procedure time would be significantly reduced, which might improve the outcomes. According to our experience, we found that several characteristics might contribute to the ability of LVIS to deal with refractory LVO, especially to deal with occlusion that was induced by hard clot [4, 7]: 1) The higher metal coverage (23%) and small cell size (< 0.9 mm) can avoid the protrusion of the hard thrombus into the vessel after stenting and angioplasty. This feature is especially suitable for treating hard clot occlusion. 2) LVIS can be full-long visualized because of the double helical tantalum strands, which is important for judging whether the stent is fully and correctly expanded. 3) Due to the closed-cell design, re-sheathability of LVIS can be up to 90%, and post-dilation balloon can be easily managed through. Based on these experiences, larger-scale clinical research was needed to confirm the efficacy and safety of LVIS in treating refractory LVO due to hard clot embolization.

Several concerns remain in dealing with refractory LVO with LVIS. Firstly, the high metal coverage and small cell size design of LVIS may lead to high re-stenosis rate [8]. In our two cases, no acute in-stent re-occlusion occurred in-hospital after stent implantation with the combination of anti-platelet drugs. In some other studies, the in-stent stenosis rate in LVIS was comparable with other stents [9]. In fact, re-stenosis is a common complication after stent implantation which can be partially avoided by anti-platelet drugs. It deserves further investigation of whether LVIS will lead to a higher re-stenosis rate after being implanted in patients with LVO. Another concern about LVIS implantation is its radial force. Due to the braided design of LVIS, its radial force may be lower than its laser-cut counterparts [4]. However, in vitro tests showed that LVIS had a comparable radial force among LEO, Enterprise, and Neuroform stents, which indicated that LVIS also has a promising radial force especially when it is fully open and pushing aggressively during the deployment [10]. At present, there is lack of data about implanting LVIS to treat LVO, and further investigation is needed to settle these concerns.

## Conclusions

In conclusion, our cases firstly revealed the feasibility of LVIS in dealing with acute ischemic stroke caused by refractory LVO due to hard clot embolization. The characteristics of LVIS give it particular advantages in treating refractory occlusion.

## Data Availability

All data generated or analyzed during this study are included in this published article.

## Abbreviations

ADAPT: A direct aspiration first-pass technique
CBF: Cerebral blood flow
DSA: Digital subtraction angiography
GCS: Glasgow Coma Scale
ICA: Internal carotid artery
LVIS: Low-profile visualized intraluminal support
LVO: Large vascular occlusion
MCA: Middle cerebral artery
mTICI: Modified Thrombolysis in Cerebral Infarction
NIHSS: National Institute of Health Stroke Scale

## References

[1] Goyal M (2016). Endovascular thrombectomy after large-vessel ischaemic stroke: a meta-analysis of individual patient data from five randomised trials. The Lancet.

[2] Chang Y (2018). Rescue stenting for failed mechanical thrombectomy in acute ischemic stroke: a multicenter experience. Stroke.

[3] Forbrig R (2019). Intracranial rescue stent angioplasty after stent-retriever thrombectomy: multicenter experience. Clin Neuroradiol.

[4] Feng Z (2016). The safety and efficacy of low profile visualized intraluminal support (LVIS) stents in assisting coil embolization of intracranial saccular aneurysms: a single center experience. J Neurointerv Surg.

[5] Munich SA, Vakharia K, Levy EI (2019). Overview of mechanical thrombectomy techniques. Neurosurgery.

[6] Li DD, et al. Solitaire stent permanent implantation as an effective rescue treatment for emergency large artery occlusion. World Neurosurg. 2019. 10.1016/j.wneu.2018.12.145 30664959

[7] Fiorella D (2016). Final results of the US humanitarian device exemption study of the low-profile visualized intraluminal support (LVIS) device. J Neurointerv Surg.

[8] Cho YD (2014). Coil embolization of intracranial saccular aneurysms using the low-profile visualized intraluminal support (LVIS) device. Neuroradiology.

[9] Feng X (2018). Comparison of recanalization and in-stent stenosis between the low-profile visualized intraluminal support stent and enterprise stent-assisted coiling for 254 intracranial aneurysms. World Neurosurg.

[10] Cho SH (2017). Bench-top comparison of physical properties of 4 commercially-available self-expanding intracranial stents. Neurointervention.

